# Effect of teriflunomide on cortex-basal ganglia-thalamus (CxBGTh) circuit glutamatergic dysregulation in the Theiler's Murine Encephalomyelitis Virus mouse model of multiple sclerosis

**DOI:** 10.1371/journal.pone.0182729

**Published:** 2017-08-10

**Authors:** Claire M. Modica, Ferdinand Schweser, Michelle L. Sudyn, Nicola Bertolino, Marilena Preda, Paul Polak, Danielle M. Siebert, Jacqueline C. Krawiecki, Michele Sveinsson, Jesper Hagemeier, Michael G. Dwyer, Suyog Pol, Robert Zivadinov

**Affiliations:** 1 Neuroscience Program, Jacobs School of Medicine and Biomedical Sciences, University at Buffalo, Buffalo, New York, United States of America; 2 Department of Neurology, Buffalo Neuroimaging Analysis Center, Jacobs School of Medicine and Biomedical Sciences, University at Buffalo, Buffalo, New York, United States of America; 3 Translational Imaging Center, Clinical and Translational Science Institute, University at Buffalo, Buffalo, New York, United States of America; 4 Exercise Science, School of Public Health and Health Professions, University at Buffalo, Buffalo, New York, United States of America; 5 Department of Geology, University at Buffalo, Buffalo, New York, United States of America; Friedrich-Alexander University Erlangen, GERMANY

## Abstract

**Background:**

Pathology of gray matter is associated with development of physical and cognitive disability in patients with multiple sclerosis. In particular, glutamatergic dysregulation in the cortex-basal ganglia-thalamus (CxBGTh) circuit could be associated with decline in these behaviors.

**Objectives:**

To investigate the effect of an immunomodulatory therapy (teriflunomide, Aubagio^®^) on changes of the CxBGTh loop in the Theiler’s Murine Encephalomyelitis Virus, (TMEV) mouse model of MS.

**Methods:**

Forty-eight (48) mice were infected with TMEV, treated with teriflunomide (24) or control vehicle (24) and followed for 39 weeks. Mice were examined with MRS and volumetric MRI scans (0, 8, 26, and 39 weeks) in the cortex, basal ganglia and thalamus, using a 9.4T scanner, and with behavioral tests (0, 4, 8, 12, 17, 26, and 39 weeks). Within conditions, MRI measures were compared between two time points by paired samples t-test and across multiple time points by repeated measures ANOVA (rmANOVA), and between conditions by independent samples t-test and rmANOVA, respectively. Data were considered as significant at the p<0.01 level and as a trend at p<0.05 level.

**Results:**

In the thalamus, the teriflunomide arm exhibited trends toward decreased glutamate levels at 8 and 26 weeks compared to the control arm (p = 0.039 and p = 0.026), while the control arm exhibited a trend toward increased glutamate between 0 to 8 weeks (p = 0.045). In the basal ganglia, the teriflunomide arm exhibited a trend toward decreased glutamate earlier than the control arm, from 0 to 8 weeks (p = 0.011), resulting in decreased glutamate compared to the control arm at 8 weeks (p = 0.016).

**Conclusions:**

Teriflunomide may reduce possible excitotoxicity in the thalamus and basal ganglia by lowering glutamate levels.

## Introduction

Multiple Sclerosis (MS) is a disease characterized by neurological disability [1] and cognitive dysfunction, [2] traditionally characterized by areas of demyelination and inflammation and brain atrophy. [3, 4] In particular, gray matter (GM) atrophy is associated with cognitive impairment, [5] and has a stronger relationship with disease progression and disability than white matter (WM) atrophy. [6] The understanding of the role of the thalamus in MS has also gained increasing interest, [3] as its volume reduction correlates with cognitive impairment [7, 8] and fatigue. [9]

These observations lead to the question of whether disparate GM regions connected by WM tracts could be pathologically affecting one another. Meningeal inflammation is present in all stages of MS, [10] and 34% [11] to 50% [12] of cortical MS lesions extend to the pial surface. Considering that apoptotic neurons are significantly increased in demyelinated cortex in MS, [12] the meninges may be involved in the pathological process which affects GM, suggesting the cortex may be one of the first GM structures involved in the disease initiation.

**Fig 1:**
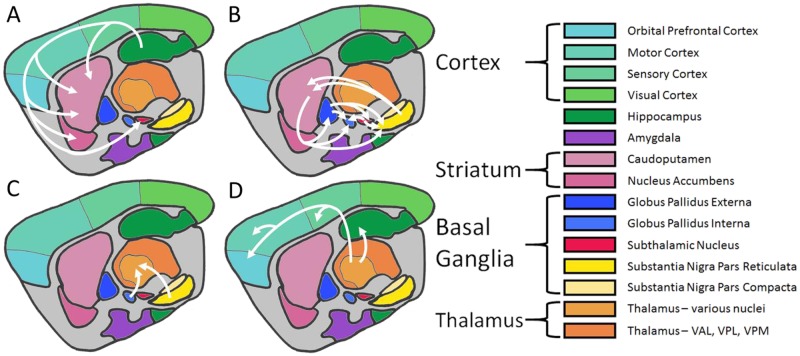
Cortex-basal ganglia-thalamus-cortex circuit in mice. Sagittal sketch of representative structures involved in the circuit. A) Cortex neurons transmit glutamate to the striatum and subthalamic nucleus. Striatum interneurons transmit acetylcholine. B) The striatum transmits GABA to the globus pallidus externa, globus pallidus interna, and substantia nigra reticulata. The globus pallidus externa transmits GABA to the subthalamic nucleus, globus pallidus interna, and substantia nigra reticulata. The subthalamic nucleus transmits glutamate to the globus pallidus interna and substantia nigra reticulata. The substantia nigra pars compacta transmits dopamine to the striatum where some terminals synapse on upregulating receptors and some on downregulating receptors. C) The globus pallidus interna and substantia nigra reticulata transmit GABA to the thalamus. D) The thalamus transmits glutamate to the cortex. While these structures and chemicals do not exist in isolation, dysregulation of any of these molecules may have reverberating effects on the entire circuit. [Figure created with information from Conn *et al*. (2005) [19], Arnsten and Rubia (2012) [51], and the Allen Mouse Brain Atlas [52].]

The cortex-basal ganglia-thalamus (CxBGTh) loop (Fig 1) is crucially influential for motor, cognitive, and affective behaviors. [13–16] For example, pathology in the substantia nigra of the basal ganglia causes reverberating problems in the CxBGTh in Parkinson’s disease. [17] Within this circuit, glutamate, an excitatory neurotransmitter, is highly regulated and linked to MS disease process in a variety of ways. MS patients with single nucleotide polymorphisms of the glutamate NMDA receptor 2A subunit domain show more advanced brain atrophy. [18] Glutamate levels, as measured by ^1^H magnetic resonance spectroscopy (MRS), are increased in acute demyelinated WM lesions. [19] Glutamate transport and metabolism are decreased in oligodendrocytes around WM lesions in MS. [20] Excitatory amino acid transporters, which typically protect neurons and oligodendrocytes from glutamate toxicity, are downregulated from the membranes of astrocytes in the demyelinated cortex containing activated microglia. [21] Therefore, dysregulated glutamate within the CxBGTh loop could lead to parallel and/or exacerbating pathology within structures in this circuit in MS patients.

We used ^1^H MRS to examine changes in the neurotransmitters glutamate and gamma-aminobutyric acid (GABA), and MRI brain volumetric outcomes, in the CxBGTh loop of Theiler’s Murine Encephalomyelitis Virus (TMEV) mice, at pre-disease baseline and after 8, 26, and 39 weeks post-induction (PI). We hypothesized that chronic meningeal inflammation, due to TMEV infection, would lead to overexcited glutamate neurons in the cortex which could increase glutamate output to the basal ganglia and thalamus, leading to increased glutamate and decreased GABA, resulting in increased inhibition of the thalamus and subsequent loss of volume in the CxBGTh loop structures.

We used TMEV infection because it produces a chronic, immune-mediated condition similar to MS. [22] The TMEV murine model is characterized by demyelination (largely in the spinal cord), neuronal death, and reduced remyelination. [23] Infected mice exhibit progressively decreasing motor coordination [23] which is associated with a development of atrophy in the brain and spinal cord. [24, 25] Iron accumulation in the thalamus, as measured by the degree of T2 hypointensity, is also associated with motor impairment. [26] Meningeal inflammation occurs throughout the chronic disease course, [27] accompanied by clusters of active IgG-producing plasma cells. [28] Therefore, the TMEV model of MS could be ideal to investigate the temporal behavior of the pathological processes in the CxBGTh loop.

Because it was shown that higher glutamate [29] and lower GABA [30] concentrations are associated with greater physical and cognitive disability and development of brain atrophy in MS patients, we chose to investigate the effect of an immunomodulatory therapy (teriflunomide, Aubagio^®^) that demonstrated a consistent effect on these outcomes in multiple clinical trials [1, 31–34] on changes of the CxBGTh loop in the TMEV mouse model of MS. Although teriflunomide is known to selectively and reversibly inhibit dihydro-orotate dehydrogenase, a key mitochondrial enzyme in the de novo pyrimidine synthesis pathway, leading to a reduction in proliferation of activated T and B lymphocytes without causing cell death, its mechanism of action is not yet fully understood. [35] In addition, previous animal experiment studies showed that teriflunomide reduced inflammation, demyelination, and axonal loss. [36]

Against this background, a control-controlled, blinded trial was designed to examine whether teriflunomide can alter temporal behavior of these pathological processes over 39 weeks. We suspected that teriflunomide could partially reverse glutamatergic dysregulation of the CxBGTh loop in the TMEV mouse model of MS because it was shown that this treatment can reduce demyelination and slowdown axonal loss. [35–37]

## Materials and methods

### Mice

Forty-eight (48) 4 to 5 week-old female SJL mice were ordered from Envigo (formerly Harlan Laboratories, Indianapolis, IN), allowed to acclimate to their new environment for one week, then given baseline behavioral testing. BHK-21 cell-purified TMEV BeAn 8386 strain working stock 20A (generously gifted from Dr. Howard Lipton, University Illinois Chicago) [38] was cultured and titrated by the Genomics/shRNA Shared Resource at Roswell Park Cancer Institute (Buffalo, NY) following published protocols. [39] 3·10^6^ PFU of TMEV was injected in 0.03mL into the right cerebrum at 7 weeks of age. Following the intraperitoneal (IP) injection of 50-100mg/kg ketamine and 2-5mg/kg xylazine anesthetic, then intracerebral injection of TMEV or saline, animals received an IP injection of 2.1mg/kg Yohimbine to counter the effects of the anesthetic. Mice were then transferred to recover under a warming lamp, and subcutaneously injected with 1-3ml of saline for recovery. Mice were monitored by technicians and laboratory staff for 1–3 days post operation to ensure stabilization. Three months after injection, at the time of peak anti-TMEV antibody levels, [40] blood was collected from mice via temporal facial vein and tested for presence of anti-TMEV antibodies; all samples were positive for infection as determined by the Mouse TMEV ELISA Kit (XpressBio, Thurmont, MD). An additional 10 sham mice injected with 0.03mL of 0.9% saline at 7 weeks of age, which were not part of the original study design but underwent behavioral testing at the same time points over a period of 39 weeks, were added to the study for post-hoc comparison purposes.

Following baseline assessments, behavioral testing was conducted on mice at 4, 8, 12, 17, 26, and 39 weeks PI and MRS/MRI examinations at 8, 26, and 39 weeks PI (S1 Fig). Time points were defined as a period of time up to 7 days before the indicated number of weeks and up to 7 days after. All behavioral, MRI and laboratory tests and analyses were conducted in a blinded manner with respect to the treatment status.

All animals were cared for and tested under The Institutional Animal Care and Use Committee (IACUC) approved protocol (NEU05124Y) by the University of Buffalo. In particular, mice were clinically monitored daily for signs of gait abnormalities, righting ability, scalding, and weight. Mice were humanely sacrificed, according to IACUC standards, by cardiac perfusion following an intraperitoneal injection of 75mg/kg sodium pentobarbital.

### Treatment

Daily administration of treatment by oral gavage began one month PI, at the time when clinical, histological, and MRI changes, and a dual surge in motor disability, suggest that acute infection shifts to chronic infection. [22, 23, 41] Teriflunomide was given to 24 mice at a dosage of 20mg/kg in 0.6% carboxymethylcellulose/0.5%, which would be equivalent to a human dose. [36] An equivalent volume of identical vehicle solution without teriflunomide was given to 23 mice (one mouse died prior to initiation of administration). The additional sham cohort of 10 saline-injected mice received no oral treatment administration.

### Behavioral tests

Clinical monitoring followed using a scale previously described. [42] Mice were evaluated for motor disability by rotarod assay (Rotamex-5, Columbus Instruments, Columbus, OH). Mice were trained on the rotarod for three days immediately preceding the baseline test. Training on the rotarod consisted of a constant speed of 3 rotations per minute (rpm) for 60s, 4rpm for 60s, and 5rpm for 60s on the first and second days, then a faux trial of the accelerating task (1 to 70rpm at an acceleration rate of 10rpm/m) on the third day. The test trial consisted of an accelerating task (1 to 70rpm at an acceleration rate of 10rpm/m). At each time point, two rotarod tests were administered, and the better of the two scores was recorded.

Mice were evaluated for cognitive decline by T-maze continuous alternation task (TCAT), consisting of free, untimed runs (periods of exploration), before returning to the start position, in a T-maze. [43] The TCAT allows the mouse to explore one arm of a T-maze per run, facilitated by blocking entry into the unexplored arm. Upon return to the start arm, the block is lifted, allowing the mouse to make a new decision of which arm to sample in the following run. Cognitive performance was quantified by the percentage of alternations, i.e. choosing to explore the arm which was not chosen in the previous run. One TCAT trial, consisting of 15 runs, was conducted at each follow-up time point, and the percentages of alternations were recorded. At baseline testing, mice were administered two TCAT trials at least 24 hours apart, and the better of the two scores was recorded.

### MRI acquisition and analysis

All MRI scanning was conducted on a 200mm horizontal-bore 9.4T magnet (Bruker Biospin, Biospec 94/20 USR) operated with ParaVision (version 5.1; Bruker Biospin) and equipped with a 440 mT/m imaging gradient system and a cryogenically cooled dual-element transceiver coil (CryoProbe, Bruker Biospin) placed over the head of the mouse. Induction and maintenance of anesthesia during imaging was achieved by inhalation of 4–5% and 1–3% Isoflurane. The total MRI scan protocol lasted approximately three hours. To prevent hypothermia, the core body temperature was continuously monitored with a rectal probe and regulated with an embedded water-heating system. Also, the probe head was heated to 37°C. Respiration rate and waveform were continuously monitored and Isoflurane concentration was adjusted if needed. In order to prevent dehydration, mice were injected with 1mL saline subcutaneously on an hourly basis. Lubricant was placed on the eyes in order to prevent drying. Respirations were monitored, and isoflurane was adjusted accordingly to maintain 20–50 breaths per minute. Following isoflurane administration for MRI scanning, mice were allowed to recover in an induction chamber under a warming lamp where they received a subcutaneous injection of 2ml of saline. Mice were then transferred under a warming lamp and subcutaneously injected with saline for recovery. In advanced infection, when clinical signs were exhibited, mice were given a small Petri plate containing recovery diet gel in addition to normal chow and subsequently monitored by technicians and laboratory staff for 1–3 days post operation to ensure stabilization.

#### MR spectroscopy

The measurement of glutamate and GABA is technically challenging, because spectroscopic peaks overlap with other metabolites, the metabolite concentrations are relatively low, they have complex multiplet resonances, and J-coupling evolutions, which impair accurate quantification with conventional MRS techniques. The special cryogenically cooled coil used in the present work enabled the direct quantification of glutamate and GABA. [44] However, while the widely employed point resolved spectroscopy (PRESS) sequence may in principle be used for this purpose, its poor spatial localization (>1mm) does not permit accurate quantification in subregions of the mouse brain as small as, e.g., the thalamus. Here, we applied an optimized ultra-short echo time stimulated echo acquisition mode (UTE-STEAM) sequence with excellent spatial localization (spatial shift less than 260μm) in the cortex, basal ganglia, and thalamus (2.6ms echo time, 2000ms repetition time, 512 averages, 6000Hz spectral width, 17min acquisition time; 1.1x1.2x2.9mm^3^ cortex voxel size, 1.5x1.2x2mm^3^ basal ganglia voxel size, 2.1x1.1x1.6mm^3^ thalamus voxel size; VAPOR water suppression with 250 Hz bandwidth). [44] The STEAM sequence provides a substantially improved spatial localization of the spectroscopic voxel compared to PRESS due to the use of 90° instead of 180° selection pulses. A transmit power gain calibration followed by a linear iterative shimming process localized in the voxel area was performed before each MRS acquisition. The combination of a high magnetic field strength (high spectral resolution), cryogenic coil (high signal-to-noise ratio) and short-TE STEAM sequence enabled the direct quantification of GABA and glutamate in small anatomical sub-regions of the mouse brain.

**Fig 2:**
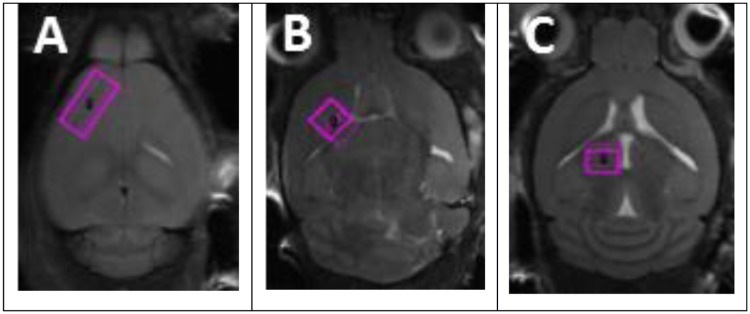
MRS voxel placement. A: cortex; B: basal ganglia; C: thalamus.

MRS was acquired in three brain regions of the hemisphere contralateral to the injection site: cortex, basal ganglia, and thalamus. MRS voxels were prescribed on dedicated high resolution localizer scans acquired immediately before the MRS (TurboRARE-T2; 3 min acquisition time). The cortex MRS voxel was placed in the prefrontal area (Fig 2A), with care used to avoid the skull and the corpus callosum. The basal ganglia voxel was placed in a volume encompassing the nucleus accumbens, caudoputamen, and globus pallidus, centered primarily in the caudoputamen (Fig 2B). The thalamus voxel was placed in the most rostral portion of the thalamus (Fig 2C), with care used to avoid WM tracts.

All spectra were analyzed using LCModel (version 6.3) [45, 46] using an appropriate basis set for the chosen sequence, phase and eddy current correction. Metabolites with a Cramér-Rao lower bound value <20% were converted into concentration ratios relative to creatine, a common internal standard in MRS [47]. To decrease the number of comparisons we examined the behavior of only glutamate and GABA over time, consistent with our original hypothesis.

#### Brain volumetry

**Fig 3:**
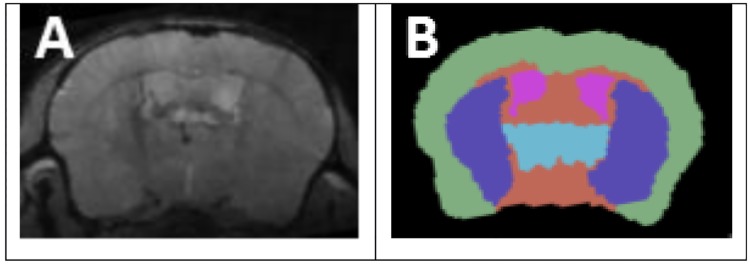
Structure segmentation. (A) Image used for segmentation. (B) Manual structure segmentation. Green: cortex; purple: basal ganglia; blue: thalamus; pink: lateral ventricles.

A multi-echo gradient echo (MEGRE) sequence was used for volumetric analysis (2.38ms first echo, 4.04ms echo spacing, 9 echoes, 90ms repetition time, one average, 18° flip angle, 27x14.3x8mm field of view, 252x180x100 matrix, 80x80x80μm resolution, 27 minutes acquisition time). MEGRE was preceded by a transmit power calibration performed in a slice (2 mm thickness) positioned at the top of the brain and a field-map based 2^nd^-order shimming to the largest possible rectangular portion of the brain while avoiding regions outside the brain. This shimming process was followed by an iterative linear shimming. For both shim configurations we assessed the FWHM of the water spectrum and used the one with the lower FWHM for the final scan. We maximized the anatomical contrast by averaging the magnitude images of all echoes (referred to as “anatomical” images in the following; Fig 3A).

For volumetric assessments, a multi-atlas approach was used. Five individual scans were selected, covering a range of atrophy levels and brain growth stages. Manual atlases were created for each of these scans by three independent operators. Regions of interest (ROIs) were outlined manually using 3D Slicer (www.slicer.org), [48] with the visual aid of Mouse BIRN Atlasing Toolkit-ready Labeled Atlas v0.6.2. [49] ROIs included the cortex, basal ganglia, thalamus, and lateral ventricles as a proxy for whole brain (Fig 3B). The cortex encompassed the entire bilateral cortical layer. The basal ganglia encompassed the bilateral caudoputamen, bilateral nucleus accumbens, and bilateral globus pallidus. The thalamus encompassed the bilateral thalamus. The lateral ventricles encompassed the bilateral most dorsal portions of the lateral ventricles.

To parcellate individual mouse images, the templates were nonlinearly aligned to the target image with ANTs (Advanced Normalization Tools) symmetric diffeomorphic image registration with cross correlation. [50] The same deformation fields were then applied to the labeled atlases corresponding to the templates to bring them into the target space. The appropriate structure label at each voxel was then selected via a joint fusion weighted voting technique between all the aligned atlases, taking into account the local voxel-wise correlations with the corresponding templates. [51] Better matching templates in each region were therefore more likely to have their atlas labels selected as the final choice.

### Tissue isolation and solochrome cyanine staining

In order to preliminarily assess the effect of TMEV infection on demyelination, coronal brain sections were stained for myelin with solochrome cyanine stain. [52] Animals (n = 5) from each treatment arm were intracardiacally perfused with saline, followed by 4% PFA after the last timepoint scan at 39 weeks. The extracted mouse brain was treated with 6 and 15% sucrose gradient, flash frozen in dry ice bath and cryosectioned into 16μm thick coronal sections. Briefly, for solochrome cyanine staining, [52] the sections were placed in Eriochrome cyanine solution (0.4% ferric chloride, 0.5% H2SO4 and 0.2% Eriochrome Cyanine RS (Sigma) in distilled water) for 15 mins. Following this, the stain was differentiated using 5.6% ferric chloride solution (in distilled water) in 30–60 second intervals up to four times, each followed by a washing step with tap water, until the WM regions were distinctly labelled blue. The images were acquired on a Zeiss Observer.Z1 microscope using 10x objective and a monochromatic camera. The images were pseudo-colored using ImageJ software [53] to restore the original stain color and emphasize the lesion sites.

### Statistical analysis

Within each treatment arm, clinical scores were compared between two time points by Wilcoxon paired samples test and across multiple time points by Friedman test; between treatment arms, clinical scores were compared at each time point by Mann Whitney U test and over multiple time points by split-plot rmANOVA. Within each treatment arm, rotarod, TCAT, volume, glutamate and GABA values were compared between two time points by paired samples t-test and across multiple time points by rmANOVA, and between treatment arms at each time point by independent samples t-test and over multiple time points by split-plot rmANOVA. Relationships between change in MRI or MRS and behavioral outcomes were analyzed by Pearson correlation. Variables determining absolute value change between 0 weeks and 39 weeks were calculated for brain volume, glutamate, and GABA. Pearson correlation coefficients between these metrics and the final clinical score, rotarod time, or TCAT percentage at 39 weeks were determined.

Data were considered as significant at the p<0.01 level and as a trend at p<0.05 level using two-tailed tests to minimize spurious findings.

## Results

### Clinical, motor, and cognitive test findings

One control mouse was removed from the analysis entirely, due to missing most MRI and clinical time points, bringing the number of control mice down to 23. One additional control mouse died after baseline during induction procedure; one teriflunomide mouse and one control mouse died between the 8 and 26 week time points; one teriflunomide and one control mouse died between the 26 and 39 week time points.

Table 1 provides comparisons of clinical, rotarod, and TCAT scores across treatment arms for each time point (clinical scores: Mann Whitney U test; rotarod, TCAT: independent samples t-test). Split-plot rmANOVA found no differences between treatment arms for change in clinical, rotarod, and TCAT scores over all time points or between any two time points.

**Table 1:** Clinical, rotarod and TCAT findings.

	Weeks	Teriflunomide (n = 24)		Control (n = 23)		Sham (n = 10)		p value(Teri vsControl)	p value(Teri vs Sham)	p value(Control vs Sham)
		Mean	SD	Mean	SD	Mean	SD			
**Clinical**	**0**	0	0	0	0	0	0	1.000	1.000	1.000
	**4**	0.03	0.08	0.05	0.10	0	0	0.596	0.077	*0*.*031*
	**8**	0.27	0.26	0.30	0.30	0.07	0.16	0.827	*0*.*005*	*≤0*.*001*
	**12**	1.04	0.95	0.78	0.38	0	0	0.334	*≤0*.*001*	*≤0*.*001*
	**17**	1.53	1.00	1.31	0.58	0.03	0.08	0.418	*≤0*.*001*	*≤0*.*001*
	**26**	1.74	0.93	1.89	0.78	0	0	0.327	*≤0*.*001*	*≤0*.*001*
	**39**	2.47	1.21	2.80	1.48	0	0	0.716	*≤0*.*001*	*≤0*.*001*
**Rotarod**	**0**	79.4	27.1	79.0	26.3	97.8	33.9	0.959	*0*.*005*	*0*.*005*
	**4**	65.8	26.7	64.3	23.7	78.5	21.3	0.843	*0*.*010*	*0*.*004*
	**8**	77.9	20.0	84.6	31.3	82.1	17.4	0.388	0.178	0.753
	**12**	88.5	34.9	89.8	32.2	81.7	22.1	0.905	0.508	0.399
	**17**	83.9	29.0	92.4	26.4	76.3	38.3	0.313	0.507	0.117
	**26**	83.6	42.3	82.0	44.4	91.5	18.8	0.908	0.828	0.732
	**39**	77.2	49.3	74.8	56.8	86.3	26.8	0.894	0.208	0.194
**TCAT**	**0**	71.0	10.2	67.4	13.7	62.6	6.43	0.310	0.116	0.786
	**4**	63.5	10.7	58.7	10.7	61.9	12.8	0.134	0.696	0.297
	**8**	61.2	11.1	63.2	11.2	54.3	18.3	0.533	0.936	0.708
	**12**	62.2	11.1	61.9	10.9	54.9	6.64	0.935	*0*.*009*	*0*.*011*
	**17**	61.2	9.4	61.1	8.9	52.8	13.3	0.960	0.169	0.175
	**26**	57.0	9.4	53.0	12.5	55.0	12.6	0.242	0.613	0.575
	**39**	57.1	12.1	51.3	10.2	56.4	13.6	0.099	0.679	0.061

Clinical is given in scores, rotarod in seconds, and TCAT in percentage. The TCAT T shape provides the mice with an untimed opportunity to explore left or right arms. Upon selection of an arm the unexplored arm was blocked off to return mouse to start. 15 trials were performed per session and left/right (L/R) arm selection was noted. Scores were determined at a percent ratio over 15 trials, with 1 for alternation and 0 for repetition. The statistical analysis was performed using Mann Whitney U test for clinical sores and independent samples t-test for rotarod and TCAT values. One control mouse was removed from the analysis entirely, due to missing most MRI and clinical time points, bringing the number of control mice down to 23. One additional control mouse died after baseline during induction procedure; one teriflunomide mice and one control mouse died between the 8 and 26 weeks times points; one teriflunomide and one control mice died between the 26 and 39 weeks’ time points.

Both the control and teriflunomide arms, compared to sham arm, showed higher clinical scores already at 4 weeks PI, which became significant at 8 weeks, and the differences remained significant at all time points throughout 39 weeks. Clinical score increased for teriflunomide and control mice across time (p<0.001, Friedman test). From 4 weeks to 39 weeks, clinical scores increased between each time point within each condition (p<0.01, Wilcoxon), with the exception of 17 to 26 weeks in teriflunomide mice, which did not exhibit a significant deterioration (p = 0.097, Wilcoxon). We observed that both teriflunomide and control cohorts were rendered spastically paralyzed with an obvious loss in posture by the end of 39 weeks. Both teriflunomide and control mice presented a waddling gait with a lowered rear at 4–8 weeks, and severe waddling gait and eventual hind limb spastic paralysis progressed over the 39 weeks of TMEV infection. Further, the sham arm did not exhibit any severe clinical disability during the study, though a slight lowering of the tail and rear end was observed in some cases at 26 and 39 weeks

Rotarod scores did not change for either teriflunomide or control condition as indicated over time by rmANOVA or between time points by paired samples t-test. However, there were several trends in both arms indicating a drop in score between 0 and 4 weeks (control p = 0.055; teriflunomide p = 0.038, paired samples t-test) and an increase in score between 4 and 8 weeks (control p = 0.023; teriflunomide p = 0.054). At baseline and at 4 weeks, both teriflunomide and control arms showed lower rotarod score, compared to the sham arm.

TCAT scores decreased in both conditions over time (p<0.01, rmANOVA), while post-hoc paired samples t-tests did not detect any changes between any two time points in either condition. However, there was a trend toward decreasing TCAT values between 0 and 4 weeks in both control (p = 0.019) and teriflunomide (p = 0.038, paired samples t-test) arms. There was also a trend toward decreasing TCAT values between 17–26 weeks in control (p = 0.035), but not in the teriflunomide arm (p = 0.225, paired samples t-test). Over 39 weeks, no TCAT differences were observed between teriflunomide and control arms compared to the sham one, except a decreased value in the sham arm at 12 weeks.

### Glutamate findings

**Fig 4:**
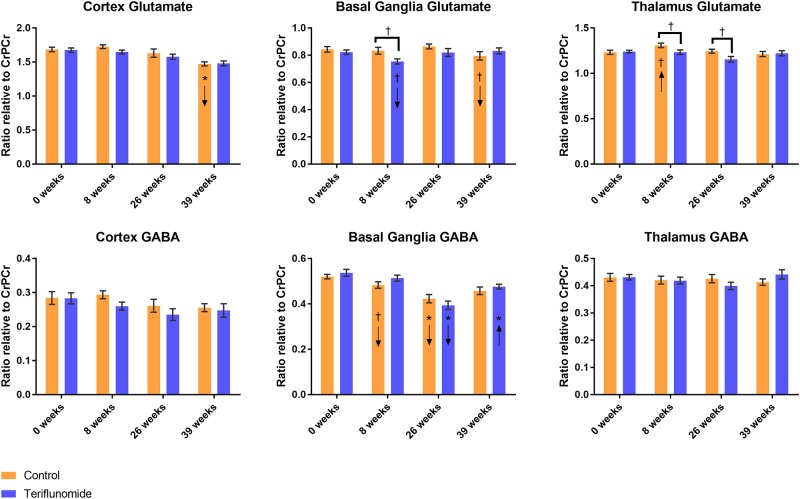
Glutamate and GABA change over time across conditions. A-C: glutamate; D-F: GABA. Brackets indicate independent samples t-test at a single time point; arrows in bars indicate direction of change from previous time point in paired samples t-test: *p<0.01, †p<0.05; orange = control, blue = teriflunomide.

Fig 4 shows glutamate ratios relative to creatine and phosphocreatine concentration and independent samples t-test p-values across treatment arms at each time point. No significant differences were detected with regard to glutamate concentrations over all time points using rmANOVA or between any two time points between treatment arms, and independent sample t-tests found no differences between treatment arms at any single time point.

#### Cortex

Glutamate in the cortex decreased over time within each treatment arm (p<0.001, rmANOVA) (Fig 4A). In control mice, this was largely driven by a decrease between 26 to 39 weeks (p<0.001, paired samples t-test), while in teriflunomide mice, the decrease was gradual across time with no significant changes between any time points as indicated by paired samples t-test.

#### Basal ganglia

There were no changes over time in either treatment arm with regards to glutamate in the basal ganglia, though the control arm trended toward decrease between 26 to 39 weeks (p = 0.033) while the teriflunomide arm trended toward decrease between 0 to 8 weeks (p = 0.011, paired samples t-tests), which resulted in a trend toward difference between the two treatment arms at 8 weeks (p = 0.016, independent samples t-test, Fig 4B).

#### Thalamus

While there were no changes over time in either condition with regards to glutamate concentration in the thalamus, there was a trend toward change in the control arm (p = 0.034, rmANOVA), largely driven by a trend toward increase between 0 to 8 weeks (p = 0.045, paired samples t-tests), resulting in a trend toward increased glutamate between two treatment arms at 8 and 26 weeks (p = 0.039 and p = 0.026, independent samples t-tests, Fig 4C).

### GABA findings

Fig 4 shows GABA ratios relative to creatine and phosphocreatine concentration and independent samples t-test p-values across treatment arms at each time point. No significant differences were detected with regard to GABA concentrations over all time points using rmANOVA or between any two time points between treatment arms, and independent sample t-tests found no differences between treatment arms at any single time point.

#### Cortex and thalamus

GABA concentrations were stable over time by rmANOVA or between time points by paired samples t-test in either treatment arm, both in the cortex and thalamus (Fig 4D and 4F). In the basal ganglia, GABA changed over time in the teriflunomide arm (p<0.001, rmANOVA), driven largely by a decrease between 8 to 26 weeks (p<0.001, paired samples t-test), followed by a significant increase between 26 to 39 weeks (p = 0.002, paired samples t-test, Fig 4E).

#### Basal ganglia

GABA in the basal ganglia only showed a trend toward change over time in the control arm (p = 0.015, rmANOVA), but this was reflected by a decrease between 8 to 26 weeks (p = 0.002, paired samples t-test) and additional trend toward decease between 0 to 8 weeks (p = 0.032, paired samples t-test). While the decrease in basal ganglia GABA from 8 to 26 weeks trended toward being larger in the teriflunomide than in the control arm (p = 0.014, split-plot rmANOVA), there was notably no subsequent increase in GABA in the control arm as indicated by paired samples t-test.

### Brain volume findings

**Fig 5:**
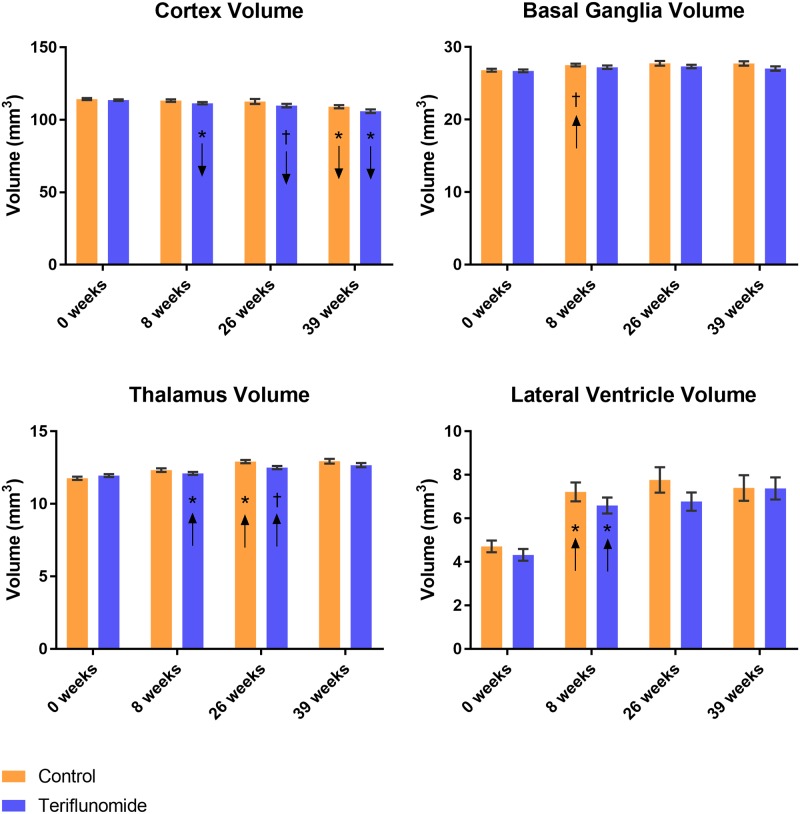
Volumetric changes over time across conditions. Arrows in bars indicate direction of change from previous time point in paired samples t-test: *p<0.01, †p<0.05; orange = control, blue = teriflunomide.

Fig 5 shows brain volume assessments of the examined structures in mm^3^ and independent samples t-test p-values across conditions at each time point. With regard to changes in brain volume measures, no significant differences between treatment arms over all time points, or between any two time points, were found by rmANOVA, and independent sample t-tests found no differences between conditions at any single time point.

#### Cortex

Volume decreased in the cortex over time in the teriflunomide condition (p<0.001, rmANOVA) (Fig 5A), and trended toward decreasing in the control condition (p = 0.013, rmANOVA). In the teriflunomide arm, this decrease was evident between 0 to 8 weeks (p = 0.006) and between 26 to 39 weeks (p<0.001), and trended toward decrease between 8 and 26 weeks (p = 0.036, paired samples t-test). In the control arm, there was a decrease only between 26 and 39 weeks (p = 0.008, paired samples t-test).

#### Basal ganglia

Brain volume did not change in either treatment arm in the basal ganglia, but the control arm trended toward increase across time and between 0 to 8 weeks (p = 0.01, rmANOVA; p = 0.025, paired samples t-test; Fig 5B).

#### Thalamus

Volume increased in the thalamus within each treatment arm (p<0.001, rmANOVA). In the control arm, this increase was driven between both 0 to 8 weeks (p<0.001) and 8 to 26 weeks (p = 0.001, paired samples t-tests, Fig 5C). These increases were not observed in the teriflunomide arm, resulting in a trend toward difference between conditions in change from 0 to 8 weeks (p = 0.02, split-plot rmANOVA), although there was a trend toward increasing between 8 to 26 weeks (p = 0.015, paired samples t-test).

#### Lateral ventricles

Volume increased in the lateral ventricles within each condition (p<0.001, rmANOVA), largely driven by an increase between 0 to 8 weeks within each condition (p<0.001, paired samples t-test, Fig 5D).

### Associations between behavioral outcomes and changes in MRI and MRS over 39 weeks

**Fig 6:**
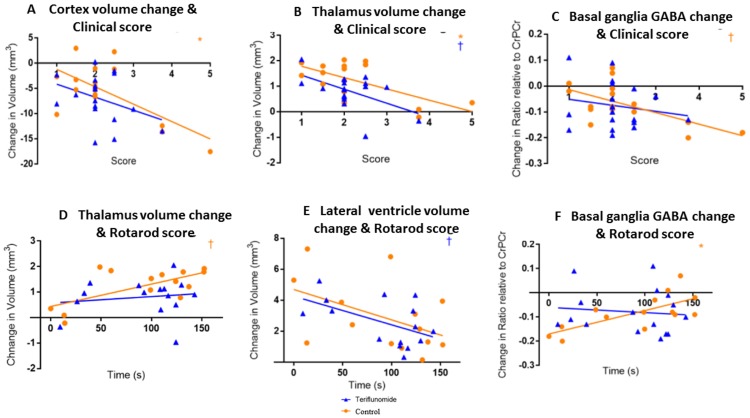
Relationships between change in MRI/MRS and clinical and rotarod score outcomes. A-C: clinical score; *p<0.01, †p<0.05; orange = control, blue = teriflunomide.

Negative associations were found between change in cortex volume (R = -0.664, p = 0.005, Fig 6A) and thalamus volume (R = -0.645, p = 0.007, Fig 6B) and clinical score in the control arm, indicating that as those volumes decreased, clinical outcomes worsened. Neither of these associations were significant in the teriflunomide arm, though one was trending (R = -0.355, p = 0.148, Fig 6A and R = -0.531, p = 0.023, Fig 6B, respectively). There were also trends toward a negative association between change in basal ganglia GABA and clinical score (R = -0.616, p = 0.011, Fig 6C). This was not observed in the teriflunomide arm (R = -0.187, p = 0.458, Fig 6C).

### Preliminary histological findings

**Fig 7:**
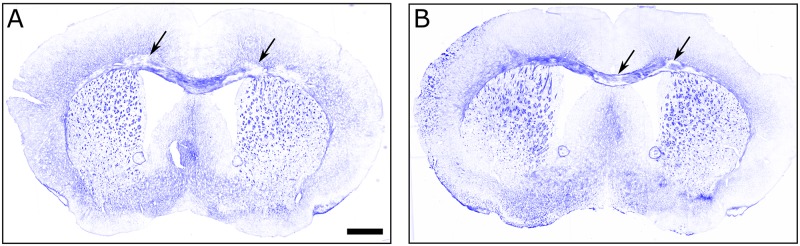
Solochrome staining TMEV induced demyelination in treated animals. A and B show images of coronal sections from teriflunomide and control animals, respectively. The arrows point at some of the demyelinated regions within the corpus callosum of each animals. Note that solochrome stain is not saturated, this is indicative of some level of demyelination even in the stained areas of the corpus callosum. The scale bar is 0.75mm long.

We detected that both control and teriflunomide treatment arms exhibited demyelinated lesions in the corpus callosum (Fig 7). We did not observe demyelination in the basal ganglia.

## Discussion

In a study of 48 mice infected by TMEV, we found that intervention with teriflunomide treatment did not significantly change the course of behavioral outcomes or MRI volumetric changes across the 39 weeks. Teriflunomide partially modulated glutamate changes in the thalamus and basal ganglia in the early/mid stage of the disease. There were different associations between clinical and MRI/MRS outcomes in the TMEV model of MS of the two treatment arms.

Mice under the treatment of teriflunomide exhibited patterns of trends toward earlier glutamate decrease in the basal ganglia. The control arm exhibited a trend toward glutamate increase in the thalamus that was not evident in the teriflunomide arm. This suggests that teriflunomide may be inducing early reduction of glutamate in the basal ganglia, and may be preventing early glutamate increase in the thalamus, possibly resulting in an overall lower concentration during the chronic infection. In combination with cross-sectional trends toward decreased concentrations of glutamate in teriflunomide arm compared to the control arm, these patterns may suggest that control mice were more likely than teriflunomide to exhibit higher levels of glutamate in the CxBGTh circuit at varying time points throughout chronic infection. These data indicate that teriflunomide intervention may help to partially promote neuroprotection by reducing possible excitotoxicity.

We found that there was increased glutamate and decreased GABA concentrations in the basal ganglia, likely leading to an environment of increased glutamate concentration in the thalamus. In line with our hypothesis, we showed that glutamate dysregulation in one of the structures of the CxBGTh could contribute to pathology in the entire circuit in the TMEV model of MS. A future histopathological analysis of the animals from the current sample (preserved) will aim to elucidate these initial MRS findings. In both treatment arms, GABA decreased between 8 to 26 weeks in the basal ganglia, however teriflunomide induced an increase in GABA in the chronic infection stage, and limited reduction of GABA in the early phase of the disease. Being an inhibitory neurotransmitter, elevated GABA may play a role in preventing excitotoxic conditions, particularly in an environment in which glutamate is elevated.

There were no volumetric differences between the two treatment arms over the follow-up. The only trend between treatment arms was evidenced between 0 to 8 weeks in the thalamus, with control arm increasing more in volume than the teriflunomide arm. These volumetric findings may be explained by greater TMEV-induced inflammation in the control arm, and by decrease of early acute inflammation in the teriflunomide arm, a phenomenon well described in MS patients, as “pseudoatrophy effect.” [54] Alternatively, increased inflammation in the control arm may have masked tissue structure loss until the later stages of chronic infection. Either scenario would also explain the trend toward increase in the basal ganglia volume exhibited by the control arm, but not the teriflunomide arm, between 0 and 8 weeks. The increase in lateral ventricle volume across all mice suggests that the infection leads to either atrophy and/or a reduction in tissue growth, and that this occurs during acute infection and/or the early part of chronic infection. Teriflunomide intervention also led to an earlier pattern of cortical volume decrease, with the teriflunomide arm decreasing, or trending toward decrease, between every time point but the control arm decreasing only between the final time points. A preliminary histological analysis confirmed demyelinated lesions in the corpus callosum, but not in the basal ganglia. More sophisticated histological analysis on the effect of the teriflunomide on myelination levels, oligodendrocyte density, maturity and microglial density will be presented in the future.

While both control and teriflunomide treatment arms exhibited significantly higher clinical progression compared to the sham arm, starting from 4–8 weeks of the follow-up, they showed similar clinical progression. It is notable that between 17 and 26 weeks, the teriflunomide arm did not exhibit significant progression while the control arm did. This was also the only pair of time points within the chronic infection period in which the control arm trended toward decline in TCAT performance while the teriflunomide arm exhibited no decline. While teriflunomide did not prevent disease progression, it may have slowed it down, particularly during this time period. We chose one month PI to begin administration of teriflunomide, however, it may be more effective to begin administration of an immunomodulatory agent at clinical sign onset, around two months PI. In a previous study we conducted, [55] we used RT-PCR to quantify TMEV viral titer in the brain and spinal cord and, even though our sample was small, we found at 21 days PI that the spinal cord-to-brain proportion of virus (0–10 fold greater) was smaller than the 46–124 days PI spinal cord-to-brain proportion (10–100 fold greater) found by Trottier *et al*. (2002) using the same method. [56] This suggests that viral load may migrate from heavy in the brain and light in the spinal cord to light in the brain and heavy in the spinal cord over time. A robust immune system may be necessary up-front, during acute infection and into the early part of chronic infection, in order to combat the heavy viral load in the brain, while an effort to decrease chronic inflammation would have greater beneficial effect after the viral titer has declined.

The trend toward decline in TCAT performance in the control arm may be the first evidence suggesting cognitive decline within the chronic infection period in TMEV, although no significant differences were found respect to the sham arm over 39 weeks. The spatial component of TCAT involves spontaneous alternation that is driven by an intrinsic motivation to seek out and explore novel space. [57] TCAT involves coordination between several brain regions including; the cortex, thalamus, substantia innominate, as well as the cerebellum. [57] For the purpose of this study, we sought to measure the general cognitive capabilities and how TMEV infection and treatment with teriflunomide affected performance, including the regions suggested previously to be involved in the TCAT task.

Rotarod testing for motor ability did not show decline in the chronic infection period, but rather a stagnation of performance, and there were no differences respect to the sham arm performance. Previous studies demonstrated an improvement of performance on similar versions of rotarod assays in healthy control mice, [24, 26] which was observed also in current study, and which could suggest that a lack of improvement in TMEV arms in our assay may be due to the effect of the infection on motor ability. Given that sham arm was not part of the original study design, and was added for post-hoc comparison purposes in a second time, future work will need to test a larger group of healthy control mice in order to determine predictable improvement patterns, or the assay may need to be modified. The assay may need to be more challenging in order to produce a better resolution between animals of slightly varying ability, or training for the rotarod assay could be more robust in order to reduce the practice effect caused by repeated follow-up testing. In addition, inclusion of more frequent MRI time points and histological analysis could characterize better acute vs. chronic stage of TMEV infection in the first 8 weeks of follow-up after PI.

These MRS and MRI findings may help to explain why cognitive and clinical scores in the teriflunomide arm did not decline in the same fashion as the control condition between 17 and 26 weeks. If teriflunomide is able to partially reduce excitotoxicity and inflammation in the CxBGTh circuit, an easing of clinical progression may just be beginning to show at 17 weeks, or three months after the start of drug administration, which is in line with human findings, indicating that it takes approximately 3 months for teriflunomide to reach the steady state. [35, 37] This is also supported by the association analyses, which found differences in associations between the two treatment arms, suggesting that teriflunomide intervention may be able to alter TMEV-induced pathology in the CxBGTh circuit and subsequent behavioral outcomes.

## Conclusions

While future studies are needed to determine the structural volume and metabolite changes of healthy control vs. TMEV-infected mice, we can conclude that teriflunomide may partially affect glutamate regulation in the TMEV model of MS, in a fashion that reduces the likelihood of early/mid-term excitotoxicity in the CxBGTh circuit. Because glutamate dysregulation is associated with MS, it is possible that teriflunomide also reduces the propensity to exictotoxicity in the CxBGTh in MS, and therefore future MRS studies on the CxBGTh circuit in MS should be conducted using a variety of disease-modifying drugs.

## Supporting information

S1 Fig Experimental timeline. MRI was conducted at 0, 8, 26, and 39 weeks. Rotarod and TCAT were conducted at 0, 4, 8, 12, 17, 26, and 39 weeks. Therapeutic intervention began at 4 weeks.
(TIF)

## References

[1] Confavreux C, Vukusic S, Moreau T, Adeleine P. Relapses and progression of disability in multiple sclerosis. The New England journal of medicine. 2000;343(20):1430–8. doi: 10.1056/NEJM200011163432001 . 11078767 10.1056/NEJM200011163432001

[2] Bobholz JA, Rao SM. Cognitive dysfunction in multiple sclerosis: a review of recent developments. Current opinion in neurology. 2003;16(3):283–8. . 12858063 10.1097/01.wco.0000073928.19076.84

[3] Zivadinov R, Havrdova E, Bergsland N, Tyblova M, Hagemeier J, Seidl Z, et al Thalamic atrophy is associated with development of clinically definite multiple sclerosis. Radiology. 2013;268(3):831–41. Epub 2013/04/25. doi: 10.1148/radiol.13122424 . 23613615 10.1148/radiol.13122424

[4] Benedict RH, Weinstock-Guttman B, Fishman I, Sharma J, Tjoa CW, Bakshi R. Prediction of neuropsychological impairment in multiple sclerosis: comparison of conventional magnetic resonance imaging measures of atrophy and lesion burden. Archives of neurology. 2004;61(2):226–30. doi: 10.1001/archneur.61.2.226 . 14967771 10.1001/archneur.61.2.226

[5] Zivadinov R, Jakimovski D, Gandhi S, Ahmed R, Dwyer MG, Horakova D, et al Clinical relevance of brain atrophy assessment in multiple sclerosis. Implications for its use in a clinical routine. Expert Rev Neurother. 2016:1–17. doi: 10.1080/14737175.2016.1181543 . 27105209 10.1080/14737175.2016.1181543

[6] Fisher E, Lee JC, Nakamura K, Rudick RA. Gray matter atrophy in multiple sclerosis: a longitudinal study. Annals of neurology. 2008;64(3):255–65. doi: 10.1002/ana.21436 . 18661561 10.1002/ana.21436

[7] Batista S, Zivadinov R, Hoogs M, Bergsland N, Heininen-Brown M, Dwyer MG, et al Basal ganglia, thalamus and neocortical atrophy predicting slowed cognitive processing in multiple sclerosis. Journal of neurology. 2012;259(1):139–46. doi: 10.1007/s00415-011-6147-1 . 21720932 10.1007/s00415-011-6147-1

[8] Schoonheim MM, Popescu V, Rueda Lopes FC, Wiebenga OT, Vrenken H, Douw L, et al Subcortical atrophy and cognition: sex effects in multiple sclerosis. Neurology. 2012;79(17):1754–61. doi: 10.1212/WNL.0b013e3182703f46 . 23019265 10.1212/WNL.0b013e3182703f46

[9] Calabrese M, Rinaldi F, Grossi P, Mattisi I, Bernardi V, Favaretto A, et al Basal ganglia and frontal/parietal cortical atrophy is associated with fatigue in relapsing-remitting multiple sclerosis. Multiple sclerosis. 2010;16(10):1220–8. doi: 10.1177/1352458510376405 . 20670981 10.1177/1352458510376405

[10] Kutzelnigg A, Lucchinetti CF, Stadelmann C, Brück W, Rauschka H, Bergmann M, et al Cortical demyelination and diffuse white matter injury in multiple sclerosis. Brain. 2005;128(11):2705–12. 16230320 10.1093/brain/awh641

[11] Lucchinetti CF, Popescu BF, Bunyan RF, Moll NM, Roemer SF, Lassmann H, et al Inflammatory cortical demyelination in early multiple sclerosis. New England Journal of Medicine. 2011;365(23):2188–97. doi: 10.1056/NEJMoa1100648 22150037 10.1056/NEJMoa1100648 PMC3282172

[12] Peterson JW, Mark S, Chang A, Trapp BD. Transected neurites, apoptotic neurons, and reduced inflammation in cortical multiple sclerosis lesions. Annals of neurology. 2001;50(3):389–400. doi: 10.1002/ana.1123 11558796 10.1002/ana.1123

[13] Minagar A, Barnett MH, Benedict RH, Pelletier D, Pirko I, Sahraian MA, et al The thalamus and multiple sclerosis: modern views on pathologic, imaging, and clinical aspects. Neurology. 2013;80(2):210–9. Epub 2013/01/09. doi: 10.1212/WNL.0b013e31827b910b ; 23296131 10.1212/WNL.0b013e31827b910b PMC3589190

[14] Cotton PL, Smith AT. Contralateral visual hemifield representations in the human pulvinar nucleus. Journal of neurophysiology. 2007;98(3):1600–9. doi: 10.1152/jn.00419.2007 . 17615131 10.1152/jn.00419.2007

[15] Haber SN, Calzavara R. The cortico-basal ganglia integrative network: the role of the thalamus. Brain research bulletin. 2009;78(2–3):69–74. doi: 10.1016/j.brainresbull.2008.09.013 . 18950692 10.1016/j.brainresbull.2008.09.013 PMC4459637

[16] Hevner RF. Development of connections in the human visual system during fetal mid-gestation: a DiI-tracing study. Journal of neuropathology and experimental neurology. 2000;59(5):385–92. . 10888368 10.1093/jnen/59.5.385

[17] Conn PJ, Battaglia G, Marino MJ, Nicoletti F. Metabotropic glutamate receptors in the basal ganglia motor circuit. Nature Reviews Neuroscience. 2005;6(10):787–98. doi: 10.1038/nrn1763 16276355 10.1038/nrn1763

[18] Strijbis EM, Inkster B, Vounou M, Naegelin Y, Kappos L, Radue EW, et al Glutamate gene polymorphisms predict brain volumes in multiple sclerosis. Multiple sclerosis. 2013;19(3):281–8. doi: 10.1177/1352458512454345 . 22851457 10.1177/1352458512454345

[19] Srinivasan R, Sailasuta N, Hurd R, Nelson S, Pelletier D. Evidence of elevated glutamate in multiple sclerosis using magnetic resonance spectroscopy at 3 T. Brain. 2005;128(Pt 5):1016–25. doi: 10.1093/brain/awh467 . 15758036 10.1093/brain/awh467

[20] Werner P, Pitt D, Raine CS. Multiple sclerosis: altered glutamate homeostasis in lesions correlates with oligodendrocyte and axonal damage. Annals of neurology. 2001;50(2):169–80. . 11506399 10.1002/ana.1077

[21] Vercellino M, Merola A, Piacentino C, Votta B, Capello E, Mancardi GL, et al Altered glutamate reuptake in relapsing-remitting and secondary progressive multiple sclerosis cortex: correlation with microglia infiltration, demyelination, and neuronal and synaptic damage. Journal of Neuropathology & Experimental Neurology. 2007;66(8):732–9. 17882017 10.1097/nen.0b013e31812571b0

[22] Oleszak EL, Chang JR, Friedman H, Katsetos CD, Platsoucas CD. Theiler's virus infection: a model for multiple sclerosis. Clinical microbiology reviews. 2004;17(1):174–207. doi: 10.1128/CMR.17.1.174-207.2004 ; 14726460 10.1128/CMR.17.1.174-207.2004 PMC321460

[23] McGavern DB, Murray PD, Rodriguez M. Quantitation of spinal cord demyelination, remyelination, atrophy, and axonal loss in a model of progressive neurologic injury. Journal of neuroscience research. 1999;58(4):492–504. 10533042 10.1002/(sici)1097-4547(19991115)58:4<492::aid-jnr3>3.0.co;2-p PMC5451093

[24] Pirko I, Johnson A, Chen Y, Lindquist D, Lohrey A, Ying J, et al Brain atrophy correlates with functional outcome in a murine model of multiple sclerosis. Neuroimage. 2011;54(2):802–6. doi: 10.1016/j.neuroimage.2010.08.055 20817104 10.1016/j.neuroimage.2010.08.055 PMC3858208

[25] Soldán P, Mateo M, Raman MR, Gamez JD, Lohrey AK, Chen Y, et al Correlation of Brain Atrophy, Disability, and Spinal Cord Atrophy in a Murine Model of Multiple Sclerosis. Journal of Neuroimaging. 2015. 10.1111/jon.12250 PMC4506196 25893491

[26] Pirko I, Johnson AJ, Lohrey AK, Chen Y, Ying J. Deep gray matter T2 hypointensity correlates with disability in a murine model of MS. Journal of the neurological sciences. 2009;282(1–2):34–8. doi: 10.1016/j.jns.2008.12.013 ; 19162280 10.1016/j.jns.2008.12.013 PMC2723804

[27] Dal Canto M, Lipton H. Multiple sclerosis. Animal model: Theiler's virus infection in mice. The American journal of pathology. 1977;88(2):497 195474 PMC2032181

[28] Pachner AR, Li L, Lagunoff D. Plasma cells in the central nervous system in the Theiler's virus model of multiple sclerosis. Journal of Neuroimmunology. 2011;232(1–2):35–40. http://dx.doi.org/10.1016/j.jneuroim.2010.09.026. 20961623 10.1016/j.jneuroim.2010.09.026

[29] Azevedo CJ, Kornak J, Chu P, Sampat M, Okuda DT, Cree BA, et al In vivo evidence of glutamate toxicity in multiple sclerosis. Annals of neurology. 2014;76(2):269–78. doi: 10.1002/ana.24202 ; 25043416 10.1002/ana.24202 PMC4142752

[30] Cawley N, Solanky BS, Muhlert N, Tur C, Edden RA, Wheeler-Kingshott CA, et al Reduced gamma-aminobutyric acid concentration is associated with physical disability in progressive multiple sclerosis. Brain. 2015;138(Pt 9):2584–95. doi: 10.1093/brain/awv209 ; 26304151 10.1093/brain/awv209 PMC4643627

[31] Miller AE, Wolinsky JS, Kappos L, Comi G, Freedman MS, Olsson TP, et al Oral teriflunomide for patients with a first clinical episode suggestive of multiple sclerosis (TOPIC): a randomised, double-blind, placebo-controlled, phase 3 trial. Lancet Neurol. 2014;13(10):977–86. doi: 10.1016/S1474-4422(14)70191-7 . 25192851 10.1016/S1474-4422(14)70191-7

[32] O'Connor P, Wolinsky JS, Confavreux C, Comi G, Kappos L, Olsson TP, et al Randomized trial of oral teriflunomide for relapsing multiple sclerosis. The New England journal of medicine. 2011;365(14):1293–303. doi: 10.1056/NEJMoa1014656 . 21991951 10.1056/NEJMoa1014656

[33] Radue E, Sprenger T, Gaetano L, Mueller-Lenke N, Wuerfel J, Wolinsky J, et al Teriflunomide slows brain volume loss in relapsing MS: a SIENA analysis of the TEMSO MRI dataset. ECTRIMS 2015;10 10, 116702.

[34] Zivadinov R, Dwyer M, Carl E, Thangavelu K, Cavalier S, Bergsland S. Evaluating the effect of teriflunomide on cortical gray matter atrophy in the phase 3 TOPIC study. AAN. 2017;P 334.

[35] Bar-Or A, Pachner A, Menguy-Vacheron F, Kaplan J, Wiendl H. Teriflunomide and its mechanism of action in multiple sclerosis. Drugs. 2014;74(6):659–74. doi: 10.1007/s40265-014-0212-x ; 24740824 10.1007/s40265-014-0212-x PMC4003395

[36] Merrill JE, Hanak S, Pu SF, Liang J, Dang C, Iglesias-Bregna D, et al Teriflunomide reduces behavioral, electrophysiological, and histopathological deficits in the Dark Agouti rat model of experimental autoimmune encephalomyelitis. Journal of neurology. 2009;256(1):89–103. doi: 10.1007/s00415-009-0075-3 . 19169851 10.1007/s00415-009-0075-3

[37] Miller AE. Teriflunomide: a once-daily oral medication for the treatment of relapsing forms of multiple sclerosis. Clin Ther. 2015;37(10):2366–80. doi: 10.1016/j.clinthera.2015.08.003 . 26365096 10.1016/j.clinthera.2015.08.003

[38] Lipton HL, Melvold R. Genetic analysis of susceptibility to Theiler's virus-induced demyelinating disease in mice. Journal of immunology. 1984;132(4):1821–5. . 6699403

[39] Jelachich M, Lipton H. Theiler's murine encephalomyelitis virus kills restrictive but not permissive cells by apoptosis. Journal of virology. 1996;70(10):6856–61. 8794327 10.1128/jvi.70.10.6856-6861.1996 PMC190733

[40] Peterson J, Waltenbaugh C, Miller S. IgG subclass responses to Theiler's murine encephalomyelitis virus infection and immunization suggest a dominant role for Th1 cells in susceptible mouse strains. Immunology. 1992;75(4):652 1350571 PMC1384845

[41] Clatch RJ, Melvold RW, Miller SD, Lipton HL. Theiler's murine encephalomyelitis virus (TMEV)-induced demyelinating disease in mice is influenced by the H-2D region: correlation with TEMV-specific delayed-type hypersensitivity. The Journal of Immunology. 1985;135(2):1408–14. 3925009

[42] Lipton HL, Kallio P, Jelachich ML. Simplified quantitative analysis of spinal cord cells from Theiler's virus-infected mice without the requirement for myelin debris removal. Journal of immunological methods. 2005;299(1–2):107–15. doi: 10.1016/j.jim.2005.01.017 . 15914195 10.1016/j.jim.2005.01.017

[43] Gerlai R. A new continuous alternation task in T-maze detects hippocampal dysfunction in mice: a strain comparison and lesion study. Behav Brain Res. 1998;95(1):91–101. 9754881 10.1016/s0166-4328(97)00214-3

[44] Bertolino N, Polak P, Preda M, Zivadinov R, Schweser F. 9.4 Tesla 1H-MRS of Glutamate and GABA in a 3.6 cubic-mm volume using an optimized UTE-STEAM sequence. Proc Intl Soc Mag Reson Med, Singapore. 2016;24:2383.

[45] Provencher S. Automatic quantitation of localized in vivo1H spectra with LCModel. NMR in Biomedicine. 2001;14(4):26–64. 10.1002/nbm.698 11410943

[46] Tkáč I, Henry PG, Andersen P, Keene CD, Low WC, Gruetter R. Highly resolved in vivo 1H NMR spectroscopy of the mouse brain at 9.4 T. Magnetic resonance in medicine. 2004;52(3):478–84. doi: 10.1002/mrm.20184 15334565 10.1002/mrm.20184

[47] Gujar SK, Maheshwari S, Björkman-Burtscher I, Sundgren PC. Magnetic resonance spectroscopy. Journal of neuro-ophthalmology. 2005;25(3):217–26. 16148633 10.1097/01.wno.0000177307.21081.81

[48] Fedorov A, Beichel R, Kalpathy-Cramer J, Finet J, Fillion-Robin J-C, Pujol S, et al 3D Slicer as an image computing platform for the Quantitative Imaging Network. Magnetic resonance imaging. 2012;30(9):1323–41. doi: 10.1016/j.mri.2012.05.001 22770690 10.1016/j.mri.2012.05.001 PMC3466397

[49] Johnson GA, Badea A, Brandenburg J, Cofer G, Fubara B, Liu S, et al Waxholm space: an image-based reference for coordinating mouse brain research. Neuroimage. 2010;53(2):365–72. Epub 2010/07/06. doi: 10.1016/j.neuroimage.2010.06.067 ; 20600960 10.1016/j.neuroimage.2010.06.067 PMC2930145

[50] Avants BB, Epstein CL, Grossman M, Gee JC. Symmetric diffeomorphic image registration with cross-correlation: evaluating automated labeling of elderly and neurodegenerative brain. Medical image analysis. 2008;12(1):26–41. doi: 10.1016/j.media.2007.06.004 17659998 10.1016/j.media.2007.06.004 PMC2276735

[51] Wang H, Yushkevich PA. Multi-atlas segmentation with joint label fusion and corrective learning-an open source implementation. Front Neuroinform. 2013;7(27):10.3389. 10.3389/fninf.2013.00027 PMC3837555 24319427

[52] Keirstead HS, Blakemore WF. Identification of post-mitotic oligodendrocytes incapable of remyelination within the demyelinated adult spinal cord. Journal of neuropathology and experimental neurology. 1997;56(11):1191–201. . 9370229 10.1097/00005072-199711000-00003

[53] Schneider CA, Rasband WS, Eliceiri KW. NIH Image to ImageJ: 25 years of image analysis. Nat Methods. 2012;9(7):671–5. . 22930834 10.1038/nmeth.2089 PMC5554542

[54] Zivadinov R, Reder A, Filippi M, Minagar A, Stüve O, Lassmann H, et al Mechanisms of action of disease-modifying agents and brain volume changes in multiple sclerosis. Neurology. 2008;71(2):136–44. doi: 10.1212/01.wnl.0000316810.01120.05 18606968 10.1212/01.wnl.0000316810.01120.05

[55] Modica CM, Sudyn ML, Zivadinov R, Pawlowski DR. Shedding Risk with Intracerebral Inoculation of Theiler’s Murine Encephalomyelitis Virus Informing a Risk Assessment. Applied Biosafety. 2016;21(3):142–50.

[56] Trottier M, Schlitt BP, Lipton HL. Enhanced detection of Theiler's virus RNA copy equivalents in the mouse central nervous system by real-time RT-PCR. Journal of virological methods. 2002;103(1):89–99. 11906736 10.1016/s0166-0934(02)00021-6

[57] Deacon RM, Rawlins JNP. T-maze alternation in the rodent. Nature protocols. 2006;1(1):7–12. doi: 10.1038/nprot.2006.2 17406205 10.1038/nprot.2006.2

