# Supplementary figures and images for: Effect of teriflunomide on cortex-basal ganglia-thalamus (CxBGTh) circuit glutamatergic dysregulation in the Theiler's Murine Encephalomyelitis Virus mouse model of multiple sclerosis
Source: PLoS One. 2017 Aug 10;12(8):e0182729. doi: 10.1371/journal.pone.0182729 (PMC5552032; doi:10.1371/journal.pone.0182729)

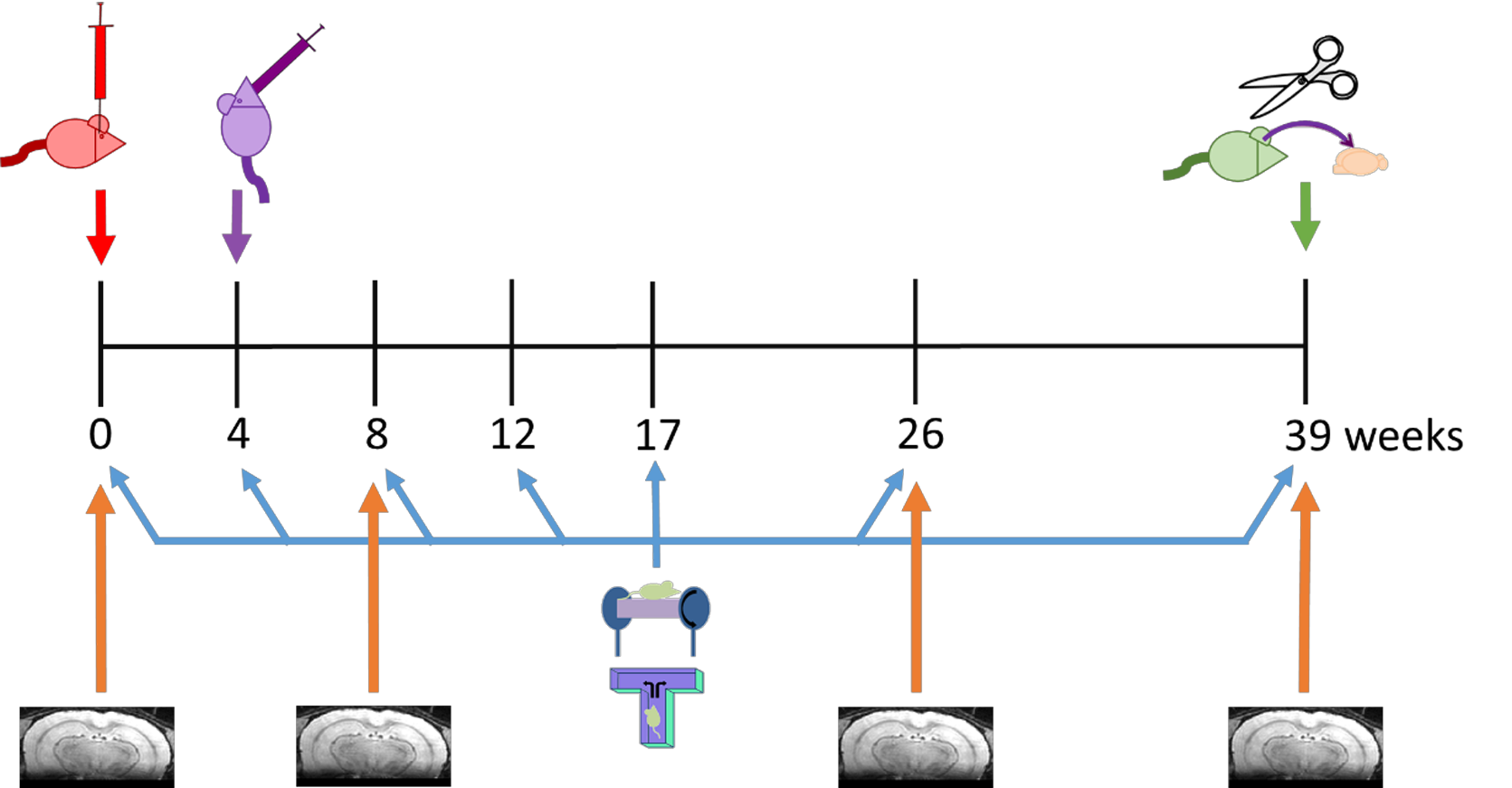

Supplement: S1 Fig — MRI was conducted at 0, 8, 26, and 39 weeks. Rotarod and TCAT were conducted at 0, 4, 8, 12, 17, 26, and 39 weeks. Therapeutic intervention began at 4 weeks. (TIF) [file pone.0182729.s001.tif]
